# Application of Theoretical Solubility Calculations and Thermal and Spectroscopic Measurements to Guide the Processing of Triamcinolone Acetonide by Hot-Melt Extrusion

**DOI:** 10.3390/pharmaceutics17050586

**Published:** 2025-04-29

**Authors:** Pedro A. Granados, Idejan P. Gross, Patrícia Medeiros-Souza, Livia L. Sá-Barreto, Guilherme M. Gelfuso, Tais Gratieri, Marcilio Cunha-Filho

**Affiliations:** 1 Laboratory of Food, Drug, and Cosmetics (LTMAC), University of Brasilia, Brasilia 70910-900, DF, Brazil; granape87@gmail.com (P.A.G.); gross.idejan@gmail.com (I.P.G.); liviabarreto@unb.br (L.L.S.-B.); gmgelfuso@unb.br (G.M.G.); tgratieri@unb.br (T.G.); 2 Department of Pharmacy, University of Brasília, Brasília 72220-900, DF, Brazil; pmedeiros@unb.br

**Keywords:** Hansen solubility parameters, hot-melt extrusion, miscibility, preformulation, thermal analysis, Fourier transform infrared spectroscopy, triamcinolone acetonide

## Abstract

**Background/Objectives**: Triamcinolone acetonide (TA), a poorly water-soluble corticosteroid, presents formulation challenges due to limited membrane permeability. This study aimed to identify suitable drug–polymer–plasticizer systems for TA using combined theoretical and experimental methods. **Methods**: Using Hansen solubility parameters, seven hot-melt extrusion (HME)-grade polymers and four plasticizers were initially screened for miscibility with TA. Based on Δδt values, four polymers—Eudragit^®^ L100 (EUD), Parteck^®^ MXP (PVA), Plasdone^®^ S-630 (PVPVA), and Aquasolve™ AS-MG (HPMCAS)—along with triethyl citrate (TEC), were selected for experimental evaluation. Differential scanning calorimetry, thermogravimetric analysis, and Fourier transform infrared spectroscopy assessed thermal behavior, miscibility, and chemical compatibility. **Results**: Amorphous TA content was highest with EUD (81.1%), followed by PVA (67.5%), PVPVA (45.6%), and HPMCAS (8.5%). Thermal incompatibility and TEC evaporation were observed in PVA, PVPVA, and HPMCAS systems. FTIR suggested TEC should be avoided in melt-based formulations with PVA and PVPVA due to PVA degradation and partial TA oxidation. No significant interactions were detected in HPMCAS samples heated to 220 °C, aligning with theoretical predictions. In contrast, the EUD–TEC system showed limited chemical reactivity and maintained TA’s structural integrity. Infrared bands at 1758 and 1802 cm^−1^ indicated minor anhydride formation above 160 °C with partial TEC evaporation. **Conclusions**: EUD/TEC were identified as a promising combination for the HME processing of TA. This work supports the rational formulation of stable amorphous systems for thermolabile drugs with poor solubility.

## 1. Introduction

Triamcinolone acetonide (TA) is an FDA-approved synthetic corticosteroid commonly used in several inflammatory conditions, such as recurrent aphthous stomatitis; oral mucositis; keloids; and hypertrophic scars, rheumatoid arthritis, and a variety of ocular diseases [1,2]. Notably, the TA angiostatic effect in the treatment of neovascularization makes this drug a potential candidate for an antiangiogenic agent that may be useful in treating various cancers [3].

Nevertheless, TA is classified as a BCS class IV drug, characterized by its low water solubility (26 µg mL^−1^) and poor membrane permeation, leading to an erratic oral bioavailability, which ends up compromising its therapeutic benefits. Therefore, pharmaceutical production strategies that can overcome such physicochemical characteristics and improve this drug’s therapeutic efficacy are required [4].

Hot-melt extrusion (HME), a solvent-free and eco-friendly approach to continuous manufacturing, appears as a technique capable of improving the solubility and bioavailability of poorly water-soluble drugs, becoming an essential tool for producing innovative pharmaceutical products [5,6]. This process is based on the miscibility of the drug within a polymer matrix by utilizing the high temperature and shear forces during extrusion. The polymeric macromolecules can stabilize the amorphous drug within the matrix by reducing its molecular mobility, consequently hindering phase segregation [7,8]. An intimate molecular mixing process can increase the drug dissolution rate [6] and, consequently, its bioavailability. Additionally, the flexibility of HME to support emerging technologies, including 3D printing, and the development of patient-specific products, further reinforces its role as a multifunctional platform in modern pharmaceutical manufacturing [6,9].

In such a promising approach, the drug–polymer interaction plays a pivotal role. Thermodynamic evaluations about drug–polymer miscibility represent a common strategy to optimize the screening of materials, reducing the cost and time of product development by HME [10,11]. Such a study involves various factors, including evaluation of drug–polymer interactions, determination of the appropriate processing temperature and screw rotation to avoid any thermal degradation, and accurate investigation of the formulation stability under different storage conditions. Additionally, recognizing critical aspects that can affect drug crystallization is indispensable in the development of drug products by HME, such as immiscibility between the drug and the polymer, enhanced mobility of the drug in the polymeric matrix due to high temperature and/or humidity, and presence of residual drug crystal or impurities that act as nucleation sites [12,13].

Furthermore, the physicochemical properties of the drug, such as its melting point, glass transition temperature, and stability profile, must be considered to tailor the HME process parameters accordingly. Through a systematic preformulation study, it is possible to effectively design HME formulations that can lead to the development of successful pharmaceutical products with enhanced therapeutic efficacy [5].

Thus, this work presents a comprehensive and innovative preformulation study to guide the processing of TA by HME. For this, materials were initially screened based on Hansen solubility parameters (HSP). Next, selected compounds were combined with TA and processed to simulate HME conditions, which were then examined using thermal and spectroscopic analyses. Particularly, the interaction between materials and drug–excipient miscibility was evaluated based on the drug’s degree of crystallinity using differential scanning calorimetry (DSC), while drug–excipient compatibility and possible deleterious interactions were assessed by thermogravimetry (TGA). Moreover, Fourier transform infrared spectroscopy (FTIR) was utilized to explore possible chemical changes or secondary interactions between the specific functional groups of the components.

## 2. Materials and Methods

### 2.1. Material

TA (C_24_H_31_FO_6_, pregna-1,4-diene-3,20-dione, 9-fluoro-11,21-dihydroxy-16,17-[(1-methylethylidene) bis (oxy)]; (11β, 16α), lot TCA-BL2701/0620) was purchased from Symbiotica Specialty Ingredients (Butterworth, Malaysia). Eudragit^®^ L100 (EUD, methacrylic acid-methyl methacrylate copolymer (1:1), lot B160703010) and Parteck^®^ MXP (PVA, polyvinyl alcohol, lot F1952064) were donated by Evonik Industries (Darmstadt, Germany) and Merck (Darmstadt, Germany), respectively. Plasdone^®^ S-630 (PVPVA, polyvinylpyrrolidone–co-vinyl–acetate, lot 002095174) and Aquasolve™ AS-MG (HPMCAS, hydroxypropylmethylcellulose acetate succinate, lot SF60G410004) were provided by Ashland Specialty Ingredients (Covington, LA, USA). The plasticizer triethyl citrate (TEC, lot S7425151) was purchased from Merck (Darmstadt, Germany). All other chemicals and solvents were of analytical grade and were used without further purification.

### 2.2. Theoretical Screening of Polymers and Plasticizers

Theoretical miscibility based on HSP was determined using seven polymers and four plasticizers widely used for HME pharmaceutical products. The miscibility of the binary combination’s polymer–drug, polymer–plasticizer, and drug–plasticizer was calculated. HSP included the total solubility parameter (δt), which consists of a vector sum of the components of Hansen’s space (Equation (1)), namely, the dispersive (δd), polar (δp), and hydrogen bonding (δh) components [14] given by Equations (2), (3), and (4), respectively:

(1) $$\delta t=\sqrt{{\delta d}^{2}+{\delta p}^{2}+{\delta h}^{2}}$$

(2) $$\delta_{d}=\frac{\sum F_{d_{i}}}{V}$$

(3) $$\delta_{p}=\frac{\sum F_{pi^{2}}}{V}$$

(4) $$\delta_{h}=\sqrt{\frac{\Sigma F_{h_{i}}}{V}}$$

where *F_di_* is the group contribution to the dispersive forces; *F_pi_* is the plane symmetry factor of polar groups; *F_hi_* is the group contribution to hydrogen bonding energy; and *V* is the molar volume [15].

The HSP values were obtained from the literature for polymers and plasticizers, while for TA, Hiroshi Yamamoto’s molecular breaking method was applied using the software HSPiP (version 5.4.08, Ipswich, UK). The TA’s simplified molecular input line entry system was obtained from the PubChem NHI database. The variation in the total solubility parameter (Δδt) was calculated by the difference between the total solubility parameter of TA and each polymer or plasticizer. Δδt was used to select materials to be subjected to further experimental tests.

### 2.3. Sample Preparation

Binary physical mixtures (BM) 1:1 (*m*/*m*) were prepared by mixing the model drug with EUD, HPMCAS, PVA, or PVPVA using a mortar and pestle for 5 min. Ternary physical mixtures (PM) of TA, individual polymers, and TEC were formulated at 5:4:1 (*m*/*m*/*m*). The PM preparation involved homogenizing the drug and polymer in a mortar and pestle for 2 min; then, the liquid plasticizer was added dropwise, and the resulting mixture was homogenized for another 3 min [5]. In addition, a fraction of the PM underwent a 5 min heating in an oven at 150 °C to simulate the thermal stress associated with the HME process [11,16]. Such samples were named heat-stressed mixtures (HM). All samples were stored in Eppendorf and aluminum bags for subsequent analysis.

### 2.4. Differential Scanning Calorimetry Analyses

Melt-quenching protocols were employed, focusing on the impact of the materials’ interaction on the TA melting point [16]. Briefly, both PM and HM samples, as well as each individual component, were subjected to heating–cooling–heating cycles in a DSC-60 instrument (Shimadzu, Kyoto, Japan) under a nitrogen atmosphere with a flow rate of 50 mL min^−1^. Approximately 2–4 mg of the samples was placed in aluminum pans. The first heating cycle (1st run) was raised to 160 °C, which is 10 °C above the glass transition temperature (*T_g_*) of EUD, the polymer with the highest *T_g_* among selected polymers. Then, the mixture was equilibrated in isothermal conditions for 2 min at 160 °C to guarantee that all polymers can form a rubbery mass with plastic properties that ensure proper flow and solubilization of the drug in the polymer matrix. The subsequent cooling cycle (2nd run) was performed to verify signs of drug recrystallization. Finally, a second heating cycle (3rd run) was carried out up to 310 °C, a few degrees above the drug’s melting point set at 294 °C. This sequence of heating and cooling cycles provides a comprehensive examination of the thermal behavior and the consequences of possible interactions within the drug–polymer system under a thermal stimulus, helping to evaluate the miscibility and potential crystallization of the drug.

The percentage of TA crystallinity was calculated based on the drug’s melting enthalpy, considering the mass fraction of TA in each mixture, i.e., 50% *m*/*m*, following Equation (5) [17].

(5) $$Drug\;crystallinity\;\left(\%\right)=\left(\frac{\Delta_{m}H}{{\Delta_{m}H}^{†}}\right)\times 100$$

where $\Delta_{m}H$ is the melting enthalpy change of TA in the mixtures and ${\Delta_{m}H}^{†}$ is the melting enthalpy change of pure TA. Analyses were performed using Thermal Analyzer software Version 2.11 (Shimadzu, Kyoto, Japan) and plotted using OriginPro 2023b software (OriginLab Corp., Northampton, MA, USA).

### 2.5. Thermogravimetric Analyses

TGA of individual compounds, BM, PM, and HM placed in platinum pans (2–5 mg) were performed by a DTG-60H (Shimadzu, Kyoto, Japan) under a nitrogen atmosphere at a flow of 50 mL min^−1^ and a heating rate of 10 °C min^−1^ from 25 to 500 °C to obtain information about the stability of the samples based on their decomposition profiles [18]. The results were compared with a theoretical profile calculated from the sum of the thermal decomposition profiles of the individual components named TM [16].

Moreover, a fraction of the PM and HM samples underwent an additional heating cycle from 25 to 220 °C to investigate the effect of the plasticizer at elevated temperatures. Next, the material was removed from the platinum pans and analyzed by FTIR. These samples were denoted as P220 and H220, respectively. Analyses were performed using LabSolutions Thermal Analyzer software (Shimadzu, Kyoto, Japan) and plotted using OriginPro 2023b software (OriginLab Corp., Northampton, MA, USA).

### 2.6. Fourier Transform Infrared Spectroscopy

FTIR analyses were performed on Bruker model vertex 70 (Billerica, MA, USA) using an ATR accessory (Billerica, MA, USA) from 4500 to 400 cm^−1^ at an optical resolution of 4.0 cm^−1^, performing 96 scans. FTIR spectrum of individual compounds and PM, HM, P220, and H220 samples were obtained. The PM spectrum was compared to the calculated average spectrum (TM) obtained by the linear combination of the pure materials normalized absorption spectra, considering the proportion of each component in the formulation [19]. The Pearson’s correlation coefficient (*R*) between all the analyzed samples was calculated using OriginPro 2023b software (OriginLab Corp., Northampton, MA, USA).

## 3. Results and Discussion

### 3.1. Theoretical Screening for Polymer and Plasticizer Selection

Although HME has been widely used to increase the solubility and dissolution of poorly water-soluble drugs by generating amorphous solid dispersions [6], the amorphous form is inherently more reactive than its crystalline counterpart and, therefore, more susceptible to thermal degradation [20]. On the other hand, while direct studies evaluating the stability of TA under HME conditions remain limited, previous studies have demonstrated drug instability in formulations produced under elevated temperatures and in the presence of trace metals [21,22]. Conversely, Hilgerot et al. [23] reported that TA exhibited thermal stability up to 190 °C when incorporated at 5% (*m*/*m*) in a polymer-drug blend used for fused deposition modeling (FDM) 3D printing. Thus, considering the scarce literature on TA stability and its possible thermal sensitivity, preformulation studies gain even more relevance in high-temperature extrusion processes.

Therefore, an initial screening of polymers and plasticizers was performed to support the development of TA dosage forms produced by HME. This first step was executed by evaluating the HSPs of the components, providing a thermodynamic estimate of their intermolecular interactions. This predictive approach facilitates the selection of polymers with optimal miscibility to the drug, thereby streamlining formulation development and reducing the need for extensive empirical screening. Nevertheless, this strategy has certain limitations, i.e., it primarily considers enthalpic contributions to miscibility while neglecting entropic effects. As a result, HSPs offer only qualitative insights into the miscibility of formulation components rather than quantitative predictions. Furthermore, HSPs do not account for the physical state of the drug after mixing with polymers or plasticizers, which can significantly impact the performance and stability of the final product [24,25].

In addition to HSP analyses, specific polymer features such as glass transition temperature, macromolecular rigidity stemming from steric hindrance, and strong polymer–polymer interactions that promote macromolecular self-association were also considered [26,27]. All these factors can affect polymer matrix mobility, influencing diffusional kinetics between components during processing [27] and, consequently, drug miscibility. All these factors can affect polymer matrix mobility, influencing diffusional kinetics between components during processing [27] and, consequently, drug miscibility.

When using HME in developing a drug product, high miscibility between the components is generally expected, i.e., the polymers should maintain the drug in the amorphous solid state while preserving its stability. The use of plasticizers, which can increase the mobility of polymer chains, is recommended to improve processability [28]. Therefore, selecting miscible materials with high interaction potential under heating conditions is a crucial step for TA processing by HME.

In this context, Table 1 presents the differences in the total Hansen solubility parameters (Δδt) between the drug, polymers, and plasticizers. These values were used to qualitatively predict the miscibility among the components, since the closer δt is between the substances, the greater the probability of the materials being miscible [1]. According to the literature, compounds with a Δδt value of 7.0 MPa^1/2^ or lower are more likely to be miscible. In contrast, compounds with a Δδt value greater than 10.0 MPa^1/2^ indicate partial miscibility, and total immiscibility is expected for those systems with Δδt > 15.0 MPa^1/2^ [29,30]. Additionally, a more stringent criterion suggests that Δδt must be below 2.0 MPa^1/2^ for the components to be considered miscible and likely to form glassy solid solutions [8,31].

Except for HPMC (Δδt = 7.9 MPa^1/2^), all polymer–drug pairs exhibited Δδt values below 7.0 MPa^1/2^, suggesting miscibility (Table 1). At the molecular level, such miscibility indicates favorable intermolecular interactions, primarily governed by dispersion and polar forces rather than hydrogen bonding, as supported by the close similarity in δd and δp values between the drug and the polymers. Nevertheless, the plasticizers, glycerin, and PPG show low miscibility with TA, as their Δδt values were 13.14 MPa^1/2^ and 8.7 MPa^1/2^, respectively. The higher Δδt values suggest weaker intermolecular affinity, likely due to significant polarity and hydrogen-bonding capacity differences, limiting adequate molecular-level mixing. In contrast, PEG400 and TEC exhibited Δδt values within the ideal miscibility range. This result implies stronger molecular compatibility, facilitating intermolecular interactions and better molecular dispersion within the polymer matrix. As TEC presented the lowest Δδt value with the drug (0.22 MPa^1/2^), it was selected for the next experimental step.

The selection of the polymers for the experimental step was based on the lowest Δδt values for polymer–drug and polymer–TEC pairs. Interestingly, among the Eudragit^®^ family polymers, E-L100 (EUD) showed the slightest difference in the Δδt values with both the drug and the plasticizer (Table 1). As a result, this polymer was chosen, together with PVPVA and PVA, as their Δδt with TEC were 1.77, 1.38, and 0.19, respectively. In addition to favorable Δδt values, these polymers were also selected due to their established use in pharmaceutical hot-melt extrusion and their distinct potential interactions with the drug stem from their specific structural and functional characteristics.

Additionally, HPMCAS was incorporated into the selection, considering its known suitability for HME processing and ability to effectively inhibit drug crystallization by forming stable solid dispersions, as demonstrated in previous studies [19,39]. Moreover, the polymer presented promising Δδt values of 4.5 MPa^1/2^ with TA and 4.72 MPa^1/2^ with TEC. Moreover, HPMCAS exhibited the closest δv value to TA, suggesting favorable volume-dependent interactions, which could help to predict compatibility under the experimental design studied in this work.

Therefore, the Δδt ranking for the expected theoretical miscibility between TA and selected polymers was PVA > EUD > PVPVA > HPMCAS. In contrast, the miscibility of TEC and polymers was as follows: PVA > PVPVA > EUD > HPMCAS.

The δh of the drug strongly differs from all the polymers and the plasticizer (Table 1), suggesting that hydrogen bonds are not the type of interaction likely to form among the materials. Indeed, previous thermodynamic assessments have revealed a significant similarity between the effect of dispersion (δd) and polarization forces (δp), contrasting with the distinct nature of the effect of δh [36]. For this purpose, if the vector sum of the δd and δp is considered, the result is the volume-dependent solubility parameter δv [36]. According to the results (Table 1), both EUD and HPMCAS δv values are very and equally close to TA, while δh of TA is closer to EUD than other polymers. Thus, more effective interactions with TA are expected for EUD.

Both HPMCAS and PVA are self-associated polymers, which means they can form strong intramolecular and intermolecular interactions within themselves, in these cases, especially by hydrogen bond formation [40]. Therefore, although the δt between PVA and TEC are very close, the polymer’s high self-association can prevent this molecule from permeating the polymer–polymer hydrogen bonds to plasticize the polymer efficiently. At the molecular scale, this self-association creates regions of polymer–polymer clustering, reducing the available free volume and limiting the diffusion of drug and plasticizer molecules. From a diffusional kinetics point of view, such behavior could lead to drug–plasticizer rich phases, hindering the efficient dispersion of the drug in the polymer matrix. Consequently, PVA mixtures are expected to exhibit higher proportions of the drug in the crystalline form when compared with EUD. On the other hand, this effect could be attenuated due to very low *Tg* and the crystalline melting of the PVA, which could contribute to higher mobility of the macromolecular chains, which does not occur for HPMCAS.

The PVPVA is a linear random copolymer of vinylpyrrolidone and vinylacetate in the proportion of 60:40, respectively. The vinylacetate monomers are inserted to reduce the *Tg* compared to the pure PVP. In this proportion, the intramolecular rigidity of this polymer is high, not due to strong self-association like for PVA and HPMCAS but due to the chemical backbone structure of the vinylpyrrolidone monomers with a ring [40,41]. Therefore, the miscibility of the drug in the PVPVA matrix is expected to be much lower than that of the systems with PVA and EUD. PVPVA tends to better interact with molecules that can act as strong hydrogen bond donors, as previously observed for riluzole drug, where the most prominent interactions occur between NH_2_ of the drug and COO and pyrrolidone carbonyl of the PVPVA [40].

Predictions based only on solubility parameters must be complemented with experimental procedures, such as thermal and spectroscopic methods, performed in the following phase of the study.

### 3.2. Differential Scanning Calorimetry Analyses

In the context of HME, considering the polymer comes into direct physical contact with the drug without any solvent, the molecules’ mobility is reduced depending on processing conditions. These distinctive features mean that results derived from traditional miscibility prediction methods, such as film-casting, are often overestimated [5]. Otherwise, thermal methods such as DSC should be a more reliable approach for better understanding miscibility in drug–polymer systems intended for processing.

Miscibility in HME compounds can be evaluated using DSC by observing the decrease in crystallinity of the drug due to drug–polymer interactions, which are usually more sensitive than X-ray diffraction [42]. Furthermore, the technique promotes conditions that favor the in situ interaction between the materials by increasing the mobility of the polymer chains above the *T_g_* or T_m_—if semicrystalline—or even by melting the drug.

**Figure 1:**
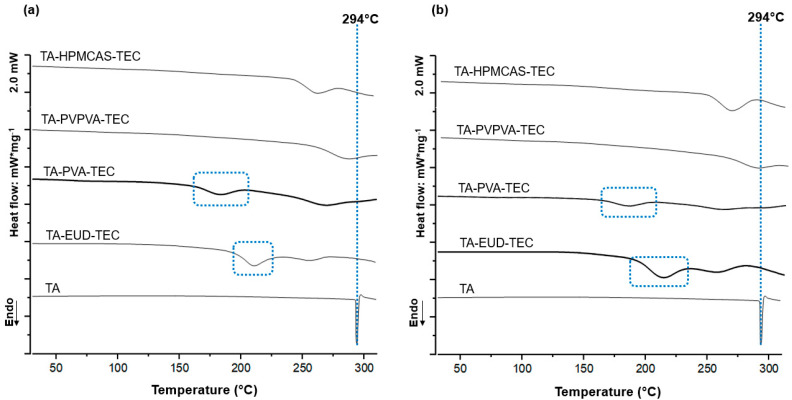
DSC curves obtained during the 3rd run of TA as supplied and its mixtures using the plasticizer TEC and the polymers EUD, PVA, PVPVA, and HPMCAS. (**a**) Ternary physical mixtures (PM); (**b**) heat-stressed mixtures (HM). The dashed blue rectangles indicate the endothermic events for EUD and PVA polymers, while the dashed blue line indicates the TA melting peak.

Thus, when analyzing the behavior of pure TA in the heating–cooling–heating cycles, no events were observed during the first run, as expected, since the highest temperature (160 °C) was below its melting point. In the second run, the DSC curve of the drug showed no exothermic peaks, suggesting no detectable recrystallization. Furthermore, during the second run, no second-order transitions were observed, indicating the maintenance of the original crystalline phase of TA. During the third run, the TA melting peak was observed at 294 °C (dashed blue in Figure 1a,b), with a corresponding melting enthalpy $\left({\Delta_{m}H}^{†}\right)$ of 156.04 J g^−1^ (Table 2), followed by decomposition. It indicates the anhydrous crystalline state of TA, often referred to as form II [43,44]. Notably, neither the first nor the second run exhibited any significant thermal events in the DSC curves of the PM or HM mixtures. Consequently, all the drug profile changes observed in the mixtures occurred due to the thermal interactions of the compounds.

The DSC curves of the samples containing EUD revealed a broad endotherm typical of the EUD polymer with a peak at 212 °C for both PM and HM samples (dashed blue rectangle in Figure 1a,b), followed by the TA melting event notably shifted to lower temperature (Table 2). The PM and HM exhibited a drug crystallinity of 15.3% (${\Delta_{m}H}^{†}$ = 23.9 J g^−1^) and 18.9% (${\Delta_{m}H}^{†}$ = 29.5 J g^−1^), respectively (Table 2). This marked reduction in the drug crystallinity in both PM and HM samples corroborates the expected high miscibility predicted by the HSP analysis for such a polymer.

Similarly, in both PM and HM containing PVA, endothermic events were observed at 183 and 185 °C (dashed blue rectangle in Figure 1a,b), respectively, possibly related to the melting of the PVA crystalline fraction, which has a semicrystalline nature [5]. The melting of the drug was shifted towards lower temperatures, occurring at 269 °C in the PM and 263 °C in the HM samples (Table 2). This temperature shift agrees with the theoretical approach and confirms the miscibility between such materials. However, the degree of interaction with PVA seems lower than with EUD based on the less pronounced reduction in crystallinity and the smaller shift of the drug’s melting peak (Table 2). This difference can be attributed to the semicrystalline nature of PVA and the strong self-associated structure of such polymer, which may hinder the complete homogenization of the drug within the polymeric matrix [45]. Interestingly, despite the known high self-association of PVA chains, their low *Tg* (Table 1), and their semicrystalline nature, which allows a crystalline melt to be achieved during the analysis temperature, could probably increase the mobility of the polymeric matrix. This phenomenon tends to attenuate the self-association tendencies of PVA, resulting in a considerable reduction in the crystallinity of the drug, as observed in Table 2.

For samples containing PVPVA, the TA melting event was shifted by 8 °C, and the percentage of drug crystallinity was slightly reduced in the PM sample compared to the pure drug (Table 2). Interestingly, the drug crystallinity was notably reduced in the HM sample (Table 2), reaching 45.6% of TA in the amorphous state, which suggests thermal treatment increases the degree of drug–polymer interaction [16]. In fact, the HM samples were subjected to conditions that allowed more time for the mixing process, probably increasing the contribution of the kinetic aspects, leading to the avoidance of the structural rigidity associated with the PVPVA chains due to the thermal excitation of their molecular segments. This finding is consistent with the predictions of the HSP, which anticipated some level of miscibility between TA and PVPVA, with less intensity than EUD or PVA.

On the other hand, both PM and HM samples containing HPMCAS showed a considerable depression in the TA melting temperature, with peaks at 267 and 268 °C, respectively (Table 2). However, the enthalpy values resulted in crystallinity of 66.1% ($\Delta_{m}H$ = 103.1 J g^−1^) for the PM and 91.5% ($\Delta_{m}H$ = 142.8 J g^−1^) for the HM sample (Table 2), indicating a strongly reduced miscibility of the drug in this system, especially after a thermal exposition. In fact, the higher crystalline fraction in the HM sample suggests that the heat treatment may induce drug recrystallization. Such a finding is also consistent with the analysis of the HSP, which located HPMCAS as the polymer with the lowest miscibility with both the drug and the plasticizer.

Notably, the proposed DSC melt-quenching protocol is effective for studying TA’s miscibility with the polymer carriers and TEC. Hence, the ability of selected polymers to maintain TA in the amorphous state after thermal stress treatment (HM samples) can be ranked as follows: EUD (81.1%) > PVA (67.5%) > PVPVA (45.6%) > HPMCAS (8.5%).

### 3.3. Thermogravimetric Analyses

**Figure 2:**
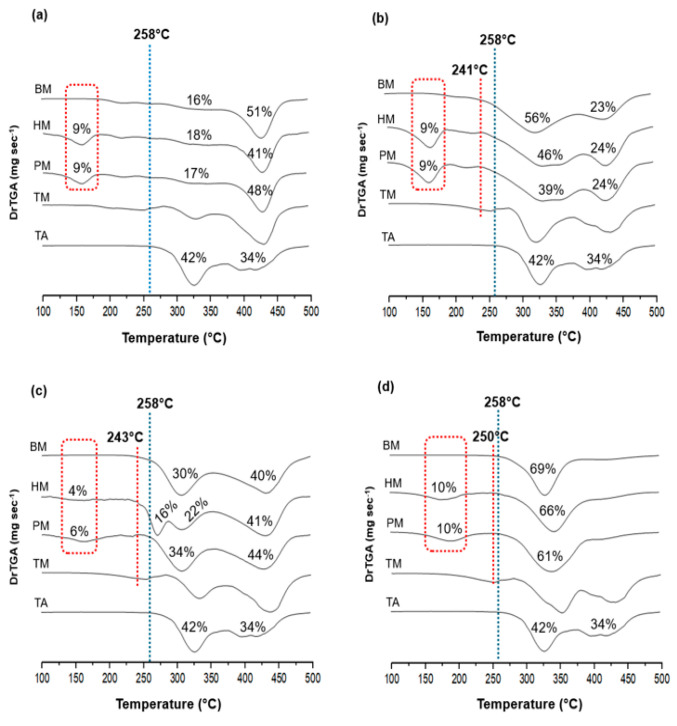
DTG curves of TA as supplied and its mixtures using the plasticizer TEC and the polymers (**a**) EUD, (**b**) PVA, (**c**) PVPVA, and (**d**) HPMCAS. The dashed blue line indicates the initial degradation event of the drug as supplied, while the dashed red line indicates the anticipation of the initial mass loss of the sample. The dashed red rectangle indicates a mass loss event related to the evaporation of TEC. The mass loss for each thermal event is represented as a percentage. TA: triamcinolone acetonide; TM: theoretical DTG curve; PM: ternary physical mixture; HM: heat stressed mixtures: BM: binary physical mixtures.

TGA curves provide valuable information about the thermal stability of the samples and possible incompatibilities among the formulation components [46]. The TGA results expressed by the DTG (Figure 2) provide a better delimitation of the mass loss steps and allow more accurate measurements of the interaction between the drug and the formulation components [47]. The decomposition onset temperature (Td) was used as a reference for comparison purposes.

TA, as supplied, presented three decomposition steps. The first with Td at 258 °C showed a mass loss of 42% (dashed blue line in Figure 2a–d), followed by a second less defined phase with an additional mass loss of 34%. The other components used in the study exhibited degradation starting at 227 °C (HPMCAS), 264 °C (PVA), 271 °C (PVPVA), and 370 °C (EUD) (Figure S1). The compatibility between the materials was assessed by comparing the theoretical (TM) and the experimental DTG profiles of PM and HM samples [16,48].

When comparing the TM with the experimental DTG curves of the samples containing PVA, PVPVA, and HPMCAS, a notable premature initial mass loss was observed in all PM and HM samples (dashed red line in Figure 2b–d), at almost the same Td for each mixture. It suggests a loss of drug stability in these samples, not necessarily related to the thermal stress treatment but due to a possible incompatibility between the components at high temperatures [5].

Notably, the HM sample containing PVPVA (Figure 2c) showed a significant change in the degradation profile, evidencing anticipation and separation of the drug degradation event into two stages. It suggests interactions between the drug and polymeric matrix may modify the thermal degradation mechanism. Remarkably, these findings align with the observed decrease in drug crystallinity following heat treatment, as evidenced by DSC analyses. Such an observation underscores the potential effectiveness of heat treatment in enhancing polymer–drug interactions; however, such interactions could potentially compromise the thermal stability of the formulation in this case.

Conversely, PM and HM samples containing EUD showed no anticipation in the initial mass loss temperature. The degradation profiles of both PM and HM samples appear to be the sum of drug and polymer behaviors (Figure 2a), indicating the thermal degradation of both drug and polymer was unaffected by each other’s presence, which suggests compatibility among the materials [16,49].

Importantly, all PM and HM samples showed a consistent initial mass loss event around 103 °C (dashed red rectangle in Figure 2a–d), probably related to TEC evaporation. Indeed, a thermal event occurring at 139 °C attributed to TEC volatilization has been documented, with mass loss increasing as its concentration increases [50]. Moreover, when analyzing pure TEC, its DTG curve revealed a mass loss between 113 and 247 °C (Figure S1). Thus, the observed variability in mass loss percentage for this event among the samples (Figure 2a–d) can be attributed to specific interactions between the plasticizer and the individual components.

A 1:1 (*m*/*m*) binary mixture (BM) was prepared by mixing TA with each polymer to corroborate this observation. None of the samples exhibited such mass loss in the temperature range expected for TEC, as indicated by the dashed red rectangle in Figure 2a–d. Of note, the anticipation of the drug degradation, previously observed for PM and HM samples containing PVA, PVPVA, and HPMCAS, was also observed for the respective binary mixtures (dashed red line in Figure 2b–d), which reinforces that may be a result of the specific drug–polymer interactions at high temperatures, near to the drug’s melting.

The evaporation of TEC observed in all the PM and HM samples is relevant since the plasticizers’ action is not limited to improving HME processing but can also significantly alter the physicochemical properties of the final product, including mechanical behavior or drug release rate. Indeed, incorporating TEC in HME processes has been shown to influence drug release profiles by modulating the physicochemical properties of the polymeric matrix, dependent on its concentration (% *m*/*m*) [51]. The evaporation of TEC could result in increased brittleness of the extrudate, potentially compromising the final formulation’s mechanical integrity. Nevertheless, several studies used TEC as a plasticizer in HME formulations processed at temperatures exceeding 180 °C [19,52]. In these cases, the authors suggested partial evaporation of the plasticizer, leading to mass loss between 0.5 and 5%, probably related to the short residence time of the material inside the extruder [19,52,53]. Such findings, together with the literature on the subject, do not invalidate its use but lead us to conclude that the use of TEC requires strict monitoring of the HME process parameters such as residence time and temperature. Alternatively, other esters from citric acid with higher boiling points and similar solubility parameters could be a successful choice to minimize such potential problems.

### 3.4. Fourier Transform Infrared Spectroscopy

**Figure 3:**
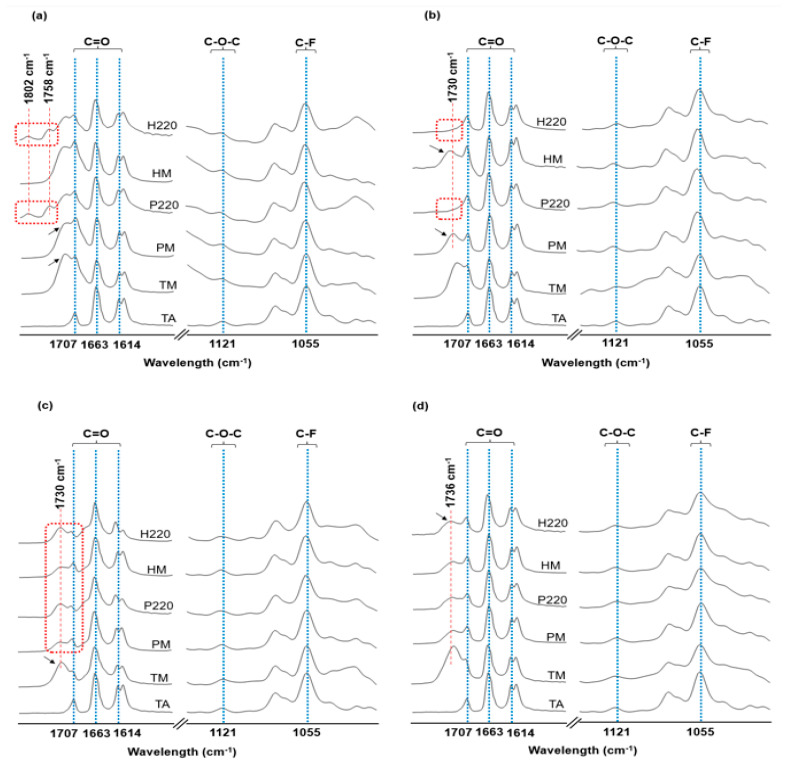
FTIR spectra of TA as supplied and its mixtures using the plasticizer TEC and the polymers (**a**) EUD, (**b**) PVA, (**c**) PVPVA, and (**d**) HPMCAS. Peaks related to TA functional groups are dashed in blue, while dashed red rectangles and lines, as well as black arrows, represent highlighted events. TA: triamcinolone acetonide; TM: theoretical FTIR spectra; PM: ternary physical mixtures; P220: PM heated up to 220 °C; HM: heat stressed mixtures; H220: HM heated up to 220 °C. The hidden-Y axis represents the arbitrary units of absorbance.

FTIR measurements were used to investigate potential chemical incompatibilities between components by observing individual functional groups and the calculated Pearson’s correlation coefficient (*R*) between the spectra [16]. In addition, both PM and HM samples were heated to 220 °C and analyzed to evaluate the presence of any residual TEC to better understand the behavior of TEC within the mixtures. Figure 3a–d show the characteristic absorption bands of the drug and their interactions with excipients in the range of 1820 to 1000 cm^−1^.

For TA, the bands that appeared in 1707; 1663; and 1614 cm^−1^ are related to the C=O stretching, while the bands at 1121 and 1055 cm^−1^ correspond to the asymmetric axial deformation of C-O-C and the vibrational stretching of C-F bond, respectively [1,54]. The plasticizer TEC (Figure S2) exhibits absorption bands in 1730 cm^−1^, corresponding to the elongation of the C=O ester bond [50]. The EUD spectra (Figure S2) showed C=O vibrations at 1721 and 1709 cm^−1^ related to the esterified carboxylic and carboxylic acid groups, respectively, as well as ester vibrations between 1255 and 1154 cm^−1^ [55]. The FTIR spectra of PVA (Figure S2) show C=O stretching related to the unhydrolyzed PVA at 1715 cm^−1^ and C-O stretching at 1141 and 1090 cm^−1^ [56]. The PVPVA infrared spectra (Figure S2) exhibit stretching vibrations of vinyl acetate C=O at 1730 cm^−1^ and C=O of amide carbonyl at 1655 cm^−1^ [57,58]. Finally, the infrared spectra of HPMCAS (Figure S2) exhibit stretching of the carbonyl group (C=O) at 1736 cm^−1^ and C-H plus C-O-C vibrations at 1375 and 1051 cm^−1^, respectively [57].

For all samples containing TA-EUD-TEC, regardless of the thermal treatment they underwent, the spectral bands corresponding to the main functional groups of the drug were recognized (dashed blue lines in Figure 3a), suggesting that their chemical structures were preserved [16]. When comparing the theoretical spectrum (TM), TA-EUD-TEC (5:4:1 *m*/*m*), to their corresponding PM spectrum, all expected bands for the drug, polymer, and plasticizer were observed. Nevertheless, the relative intensity of the absorption band at 1730 cm^−1^, probably related to the TEC, was lower in PM (arrows in Figure 3a), suggesting partial volatilization of the plasticizer; even so, it did not seem to influence the sample stability. This result is expected since the values of the δd component (Table 1) suggest that the interactions between TA, EUD, and TEC are mainly dispersive, and the *R*-value (0.990) falls within the recommended standards for the chemical similarity between samples [5]. Moreover, the similarity between the sample components was determined for the PM-HM samples, obtaining an *R*-value close to 1 (0.999), and no spectral shifts or presence of new absorption peaks were detected. This result suggests the lack of any new relevant chemical changes after thermal stress [59].

On the other hand, when comparing the PM-P220 and HM-H220 spectra (Figure 3a), the *R* values were, respectively, 0.901 and 0.899. These values were lower than those previously observed; however, correlation coefficient values above 0.85 have been reported as non-significant destructive interactions for the drug [17]. In fact, for both samples, all absorption peak characteristics of TA can be observed without relevant shifts or broadening (dashed blue lines in Figure 3a), reinforcing the drug stability at this temperature range. Nevertheless, when observing the region above 1730 cm^−1^, in both P220 and H220 sample spectra, we identified a shoulder at 1758 cm^−1^ and a new peak at 1802 cm^−1^ (dashed red rectangle in Figure 3a), probably related to the polymer and not to the plasticizer’s volatilization. As the active EUD carboxylic groups represent approximately 48% of their molecular mass [34], the formation of anhydrides intra- or intermolecularly during heating can be expected [60]. Indeed, new peaks at 1804 and 1673 cm^−1^ that gradually increased intensity on heating above 193 °C were already reported [61]. This anhydride formation was heat-dependent and increased at 200 °C [61]. Although anhydride formation is confirmed under thermal stress, this process is reversible in the presence of moisture and is unlikely to persist under standard storage conditions. The formation of anhydrides must be carefully evaluated through the thermal process since these compounds are highly reactive with nucleophiles and can acylate several critical functional groups of proteins and macromolecules [62].

Although TA contains both primary and secondary hydroxyl groups, their reactivity is limited due to their position within a rigid, polycyclic steroidal structure, and such a reaction will usually require solvent-assisted conditions. Moreover, no new peaks associated with ester bond formation were observed in the fingerprint region of the FTIR spectra, and all characteristic bands of TA remained unchanged after thermal treatment. The preservation of the spectral profile and high *R*-values further support the conclusion that TA does not undergo esterification or degradation under the tested conditions. Furthermore, it is expected that the remaining amount of plasticizer will be able to form hydrogen bonds with the polymer and generate its plasticization.

Otherwise, in all samples containing TA-PVA-TEC, regardless of the thermal treatment to which they were subjected, the main TA functional groups spectral bands were recognized (dashed blue lines in Figure 3b), suggesting that its chemical stability up to 220 °C was preserved [16]. Interestingly, when comparing the theoretical and experimental spectrum, TM-PM, the *R*-value found was 0.897. Nevertheless, the spectral bands corresponding to the TA functional groups were recognized in both samples. Furthermore, when comparing the spectral bands of both PM and HM samples, a possible overlap of the PVA C=O groups and TEC at 1730 cm^−1^ was observed (arrows in Figure 3b). When observing the same region of the spectrum in samples P220 and H220, the overlapped absorption band disappeared (dashed red rectangle in Figure 3b), suggesting that the mass loss (9%) observed in the DTG for the sample containing PVA before 220 °C (dashed red rectangle in Figure 2b) may correspond to the volatilization of TEC and the initial degradation of the polymer triggered by inherent compatibility issues derived from heating.

PVA has been proven challenging to process by melting, as its melting point is close to the decomposition temperature, where the hydroxyl and acetate side groups are eliminated to form water and acetic acid [63]. The addition of plasticizer can be used to improve the processing window; however, the presence of TEC has shown a minimal effect when the objective is to increase the segmental mobility of PVA molecules. In addition, the thermal instability of TEC was already associated with highly unstable PVA-TEC mixtures [63], suggesting that using TEC in PVA melting-based pharmaceutical formulation processes should be avoided, as it leads to thermal degradation of PVA [63]. The results are consistent with and help to elucidate the thermal events observed in the DTG curves.

For the PVPVA-containing samples, no shifts were identified between the calculated (TM) and experimental (PM) spectra (Figure 3c), and the *R*-value obtained for these samples was 0.972, which falls within the recommended standards for the chemical similarity between samples [5]. However, the absorption band at 1730 cm^−1^, probably related to the overlapping C=O stretching vibrations of the polymer and plasticizer, is more intense in the TM (arrow in Figure 3c), suggesting the partial volatilization of TEC even in the PM sample. In addition, when observing the absorption spectra of both P220 and H220 samples and compared with their respective PM and HM samples spectra, the drug C=O absorption band at 1707 cm^−1^ had a lower relative intensity as compared with the C=O absorption band of the overlapping PVPVA and TEC at 1730 cm^−1^ (dashed red rectangle in Figure 3c), suggesting the partial oxidation of the drug at these temperature ranges.

Indeed, the use of PVPVA in solid dispersions is associated with stability concerns due to trace amounts of peroxides, which may induce oxidative degradation of oxygen-sensitive drugs such as TA [21,58]. Furthermore, it has been demonstrated that mixtures containing PVPVA placed in open glass vials and stored in an oven at 125 °C for 31 days resulted in significant formation of peroxide impurities that could oxide drug substances either through a free-radical mechanism involving oxygen or direct oxidation by a peroxide [64]. As oxidation is the predominant mechanism of TA degradation [21], TGA and FTIR results suggest that using PVPVA in combination with TEC as a plasticizer for the formulation of TA at elevated temperatures should be avoided. These findings imply that oxidation processes triggered by residual peroxides in PVPVA may continue during storage, particularly under humid or elevated temperature conditions, posing a risk to both the formulation’s long-term stability and therapeutic effectiveness. Therefore, strict control of storage conditions and possibly incorporating stabilizers may be required to prevent oxidative degradation.

For all samples containing TA-HPMCAS-TEC (Figure 3d), regardless of the thermal process to which they were subjected, the spectral bands that corresponded to the drug’s main functional groups were recognized (dashed blue lines in Figure 3d), suggesting that its chemical stability up to 220 °C was preserved [16]. All expected TA, HPMCAS, and TEC bands were observed in the PM sample spectra, and no shifts were identified between the calculated and experimental spectra. Furthermore, the *R*-value between TM-PM samples (0.966) fell within the recommended standards for chemical similarity [5]. Interestingly, in all samples, regardless of the heat treatment, it is possible to observe an overlapping between the carbonyl stretching groups of polymer and plasticizer at 1736 cm^−1^ (black arrow and red dash line in Figure 3d) without changes in frequency or weakening of the bond strength. It suggests no detectable formation of hydrogen bonds or interactions between the components. Although the correlation coefficient values were higher than 0.955 in all the samples, guaranteeing the chemical stability of the drug, the lack of detectable interaction was evident since all the FTIR spectra were almost the same. It seems to agree with the results obtained in the thermal analysis once the DSC curve showed a state of high TA crystallinity within this polymeric matrix, and the TGA degradation profile was found to be the sum of the mixture components (Figure 2d). This outcome reinforces the findings of HSP, which suggest the greater difference between the Δδh values of these materials is responsible for the poorly observed miscibility. Indeed, such a polymer, although compatible, is too inert to be useful in an HME process with TA.

## 4. Conclusions

The utility of preformulation studies extends beyond merely indicating a qualitative assessment among formulation components, providing more comprehensive insights into the conformation of materials under simulated HME conditions. Such studies allow anticipation and monitoring of likely chemical and physical events, which is crucial in developing pharmaceutical formulations. In this context, integrating theoretical predictions with experimental thermal and spectroscopic techniques effectively guides the selection of suitable materials for drug products obtained by HME, allowing the rationalization of the experimental effort. Particularly, HSP has proven valuable in predicting the miscibility of systems. However, such predictions should be interpreted considering specific polymer characteristics, such as glass transition temperature, macromolecular rigidity, and strong polymer–polymer interactions. These factors can influence the mobility of the polymer and, subsequently, the diffusional kinetics between components during processing.

Hence, this study successfully utilized a theoretical approach to select materials that are more likely to yield favorable outcomes for TA processed via HME. Indeed, the experimental phase confirmed the predicted miscibility between TA-EUD-TEC and their compatibility. Nevertheless, it is crucial to carefully monitor the partial TEC evaporation and the formation of anhydrides derived from the active EUD carboxylic groups during the heating. Despite these challenges, the findings indicate that using TEC as a plasticizer in PVA and PVPVA melting-based pharmaceutical formulation processes should be avoided, as it leads to unstable PVA-TEC mixtures and partial oxidation of the drug in the PVPVA containing samples.

Overall, the outcomes of this preformulation study demonstrate that the combination of EUD and TEC holds promise for the HME processing of TA, highlighting the critical role of careful excipient selection in developing stable and effective pharmaceutical formulations. Beyond its application to TA, the methodology employed in this study may be extended to other active pharmaceutical ingredients with comparable physicochemical properties, particularly for other corticosteroids and other drugs with high melting points. Future research could focus on developing and optimizing HME formulations, including in vitro characterization, such as dissolution, mucoadhesion, and mechanical performance tests, to support their potential applications.

## Figures and Tables

**Table 1:** Theoretical miscibility between TA, polymers, and plasticizers. The total solubility parameter (δt) of each material is presented, as well as their individual δt components, namely, dispersive (δd), polar (δp), and hydrogen bonding (δh). The δv represents the volume-dependent solubility parameter, a vector sum of the δd and δp components, while the Δδt represents the difference in the total solubility parameter between the pairs drug–polymer |TA-P|, drug–plasticizer |TA-Pl|, and polymer–plasticizer |P-TEC|. The *Tg* of the polymers is also shown.

Material	δt (MPa^1/2^)	δt Components (MPa^1/2^)				Δδt (MPa^1/2^)			T*g* (°C)	Ref.
		δd	δp	δh	δv	|TA-P|	|TA-Pl|	|P-TEC|		
TA	21.2	18.7	7.7	6.4	20.22	---	---	---	---	Author
HPMC	29.1	---	---	---		7.9	---	8.12	117	[32]
HPMCAS	25.7	20.5	5.1	14.6	21.12	4.5	---	4.72	135	[19,33]
E-E100	18.9	---	---	---		2.3	---	2.08	48	[34,35]
E-L100	22.75	19.31	0.41	12.03	19.31	1.55	---	1.77	150	[34,36]
E-S100	18.38	---	---	---		2.82	---	2.6	150	[32]
PVA	21.17	11.2	12.40	13.0	16.44	0.03	---	0.19	45	[5,19]
PVPVA	19.60	0.64	18.0	18.01	7.73	1.60	---	1.38	105	[37]
Glycerin	34.34	---	---	---		---	13.14	---	---	[14]
PEG400	18.9	---	---	---		---	2.3	---	---	[38]
PPG	29.9	---	---	---		---	8.7	---	---	[14]
TEC	20.98	16.5	4.90	12.0	17.21	---	0.22	---	---	[14,19]

TA: triamcinolone acetonide; HPMC: hydroxypropylmethylcellulose; HPMCAS: hydroxypropylmethylcellulose acetate succinate; E: Eudragit^®^; PVA: polyvinyl alcohol; PVPVA: polyvinylpyrrolidone–co-vinyl–acetate; PEG400: polyethylene glycol 400; PPG: 1,2 propylene glycol; TEC: triethyl citrate; P: polymer; Pl: plasticizer.

**Table 2:** DSC interactions observed during the 3rd run of TA as supplied and its mixtures using the plasticizer TEC and the polymers EUD; PVA; PVPVA; and HPMCAS. The percentage of the drug’s crystallinity and the associated drug’s enthalpy value $\left(\Delta_{m}H\right)$ are indicated for each mixture, while the TA melting peak indicates the change in the melting temperature of the drug. ${\Delta_{m}H}^{†}$ = melting enthalpy of TA raw material. PM: ternary physical mixtures; HM: heat-stressed mixtures.

Sample	PM			HM		
	Drug Crystallinity (%)	$\Delta_{m}H$ (Jg^−1^)	TA Melting Peak (°C)	Drug Crystallinity (%)	$\Delta_{m}H$ (Jg^−1^)	TA Melting Peak (°C)
TA-EUD-TEC	15.3	23.9	255	18.9	29.5	255
TA-PVA-TEC	34.7	54.1	269	32.5	50.6	263
TA-PVPVA-TEC	96.9	151.1	287	54.4	84.9	286
TA-HPMCAS-TEC	66.1	103.1	267	91.5	142.8	268

TA raw material: drug crystallinity = 100%; ${\Delta_{m}H}^{†}$ = 156.04 J g^−1^; melting peak = 294 °C.

## Data Availability

The data presented in this study are available in this article.

## Supplementary Materials

The following supporting information can be downloaded at https://www.mdpi.com/article/10.3390/pharmaceutics17050586/s1. Figure S1. Thermogravimetric curves represented by the first thermogravimetric derivative of the polymers as supplied EUD, PVA, PVPVA, and HPMCAS, and the plasticizer TEC; Figure S2. FTIR spectra of the polymers as supplied EUD, PVA, PVPVA, and HPMCAS, and the plasticizer TEC. The hidden-Y axis represents the arbitrary units of absorbance.

## Abbreviations

The following abbreviations are used in this manuscript:

TA	Triamcinolone acetonide
HME	Hot-melt extrusion
HSP	Hansen solubility parameters
EUD	Eudragit^®^ L100
PVA	Parteck^®^ MXP
PVPVA	Plasdone^®^ S-630
HPMCAS	Aquasolve™ AS-MG
TEC	Triethyl citrate
DSC	Differential scanning calorimetry
TGA	Thermogravimetry
FTIR	Fourier transform infrared spectroscopy
BM	Binary physical mixtures
PM	Ternary physical mixtures
HM	Heat-stressed mixtures
Tg	Glass transition temperature
TM	Theoretical profile
P220	PM with additional heating cycle
H220	HM with additional heating cycle
