# Supplementary material for: Application of Theoretical Solubility Calculations and Thermal and Spectroscopic Measurements to Guide the Processing of Triamcinolone Acetonide by Hot-Melt Extrusion
Source: Pharmaceutics. 2025 Apr 29;17(5):586. doi: 10.3390/pharmaceutics17050586 (PMC12114760; doi:10.3390/pharmaceutics17050586)
Supplement: Supplementary file 1 [file pharmaceutics-17-00586-s001.zip › pharmaceutics-3582438-supplementary.pdf]

Supplementary Materials

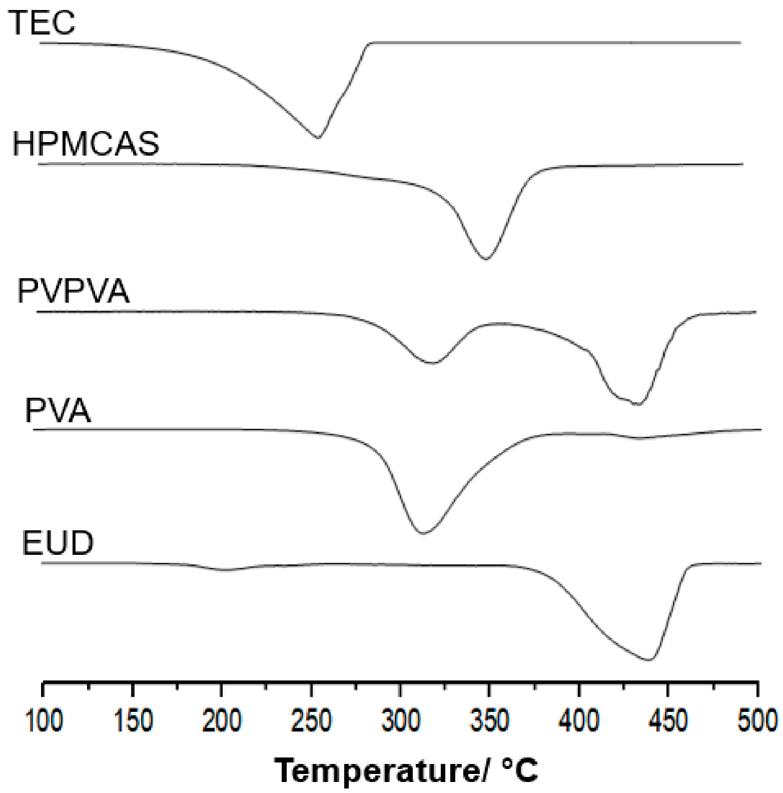

Figure S1 Thermogravimetric curves represented by the first thermogravimetric derivative of the polymers as supplied EUD, PVA, PVPVA, and HPMCAS, and the plasticizer TEC.

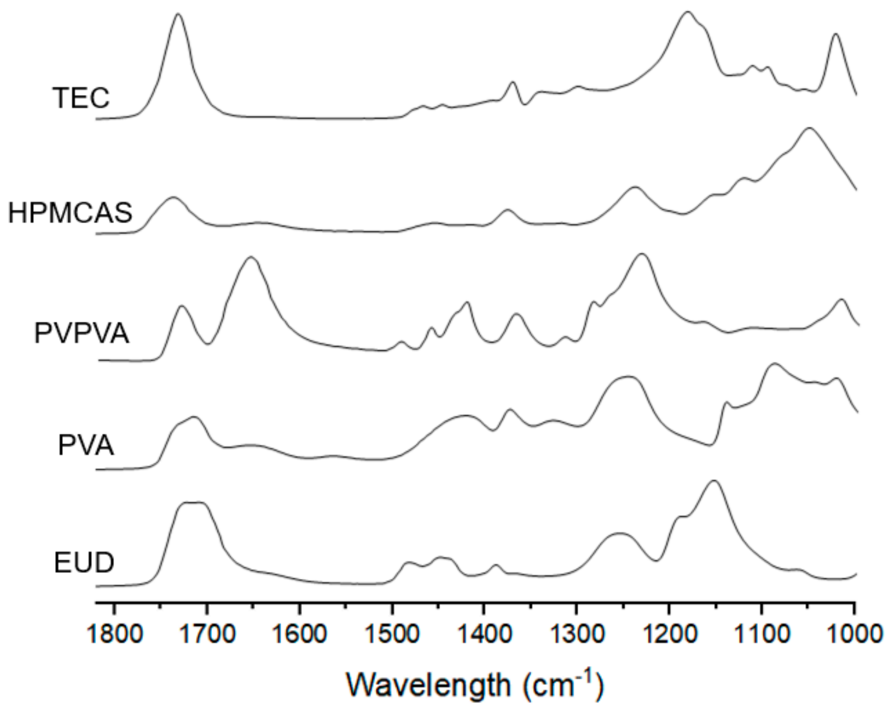

Figure S2 FTIR spectra of the polymers as supplied EUD, PVA, PVPVA, and HPMCAS, and the plasticizer TEC. The hidden-Y axis represents the arbitrary units of absorbance.
